# Light sensors for objective light measurement in ambulatory polysomnography

**DOI:** 10.1371/journal.pone.0188124

**Published:** 2017-11-16

**Authors:** Rachel Schembri, Jo Spong, Allison Peters, Peter Rochford, Philip Wilksch, Fergal J. O’Donoghue, Kenneth M. Greenwood, Maree Barnes, Gerard A. Kennedy, David J. Berlowitz

**Affiliations:** 1 The Institute for Breathing and Sleep, Austin Health, Heidelberg, VIC, Australia; 2 Department of Medicine, Dentistry and Health Sciences, The University of Melbourne, Parkville, VIC, Australia; 3 College of Science, Health and Engineering, La Trobe Rural Health School, La Trobe University, Bendigo, VIC, Australia; 4 Melbourne Sleep Disorders Centre, Melbourne, VIC, Australia; 5 School of Science, RMIT University, Melbourne, VIC, Australia; 6 James Cook University, Singapore, Singapore; 7 School of Health and Biomedical Sciences, RMIT University, Melbourne, VIC, Australia; University of L'Aquila, ITALY

## Abstract

Ambulatory polysomnography (PSG) does not commonly include an objective measure of light to determine the time of lights off (Loff), and thus cannot be used to calculate important indices such as sleep onset latency and sleep efficiency. This study examined the technical specifications and appropriateness of a prototype light sensor (LS) for use in ambulatory Compumedics Somte PSG.Two studies were conducted. The first examined the light measurement characteristics of the LS when used with a portable PSG device, specifically recording trace range, linearity, sensitivity, and stability. This involved the LS being exposed to varying incandescent and fluorescent light levels in a light controlled room. Secondly, the LS was trialled in 24 home and 12 hospital ambulatory PSGs to investigate whether light levels in home and hospital settings were within the recording range of the LS, and to quantify the typical light intensity reduction at the time of Loff. A preliminary exploration of clinical utility was also conducted. Linearity between LS voltage and lux was demonstrated, and the LS trace was stable over 14 hours of recording. The observed maximum voltage output of the LS/PSG device was 250 mV, corresponding to a maximum recording range of 350 lux and 523 lux for incandescent and fluorescent light respectively. At the time of Loff, light levels were within the recording range of the LS, and on average dropped by 72 lux (9–245) in the home and 76 lux (4–348) in the hospital setting. Results suggest that clinical utility was greatest in hospital settings where patients are less mobile. The LS was a simple and effective objective marker of light level in portable PSG, which can be used to identify Loff in ambulatory PSG. This allows measurement of additional sleep indices and support with clinical decisions.

## Introduction

Comprehensive assessment of sleep and breathing requires full polysomnography (PSG), however, in-laboratory PSG evaluates typical sleep in an atypical environment. Portable PSG attempts to overcome these issues of validity by measuring sleep in the typical environment for that person. Portable PSG is usually unattended, lowering staff costs while improving patient access, maintaining usual care provision for those with illness or disability, and avoiding the unfamiliar environment of a sleep laboratory. [1–4]

In-laboratory PSG signals commonly include a measure of light, however portable PSG do not commonly include a light sensor (LS) and thus the time of lights off (Loff) is unable to be objectively determined. Without accurate light data, important indices such as sleep onset latency and sleep efficiency (SE) cannot be reliably determined. Light level intensity varies significantly between environments where PSG is undertaken (for example, in homes or on hospital wards). While the absolute intensity, as measured in lux, is arguably not necessary, determining when a person turns off the light in readiness for sleep (the “moment” of Loff) would improve the accuracy of many sleep indices derived from ambulatory PSG.

This study evaluated a prototype LS used with an ambulatory PSG device in both home and hospital ward settings. Two assessments were conducted. The first “bench test” examined the light measurement characteristics of the LS to determine the range, sensitivity, linearity and the effect of changes in light source type. The second “real-world test” investigated LS recording ranges in home and hospital settings. The aim of these studies was to validate the LS for use in ambulatory PSG.

## Materials and methods

### Experimental equipment

The LS photosensor is a PerkinElmer Optoelectronics VTB8440B planar silicon photodiode with a parallel resistor (220 kΩ). Apart from a modification to the LS connection such that it fitted the auxiliary DC port of the portable recording unit, all other aspects of the LS remained the same as the commercially available, in-laboratory version (Compumedics model 7707-0030-01, Abbotsford, Victoria, Australia). The light sensor is a small light weight sensor, approximately measuring 1.5cm x 2cm and weighing 3g. LS traces were recorded and analysed via portable Compumedics ‘Somte PSG’ devices and Compumedics ‘Profusion 3’ software (Compumedics, Abbotsford, Victoria, Australia). The Somte PSG auxiliary port had a maximum input range of +/-250mV.

### Procedure

#### Study 1: Light measurement characteristics of the light sensor

The LS was placed in a room where light levels could be varied to known lux levels and light recorded across a range of lux. Three LS and Somte PSG recording unit pairs (LS/Somte PSG) were used. Lux was measured with a digital EC1 Luxmeter (Hagner, Sweden) which incorporated a silicon photodiode with a wavelength filter and diffuser. The luxmeter had accuracy within 1% and a resolution of 0.1 lux. To achieve light levels above and below 180 lux, two incandescent light sources were used: a 150 watt tungsten filament flood lamp and a 50 watt quartz-halogen bulb, respectively. To adjust light levels across the experimental range, neutral absorbing filters with optical densities of 1.0 and 2.0 (transmittances of 0.1 and 0.01 respectively) and varying distances between the LS and the light source (20 cm to 4 m) were used.

For each of the three LS/Somte PSG pairs, 30 seconds of light recording data (sampling rate of 32 Hz) were averaged to provide one voltage value per light level sampled. The upper light limit of the LS/Somte PSG pairs was found by exposing the sensor to bright light (1000 lux) and reducing lux in increments of 100 until the recorded voltage dropped below the maximum. Linearity was then assessed by recording a range of light levels from this upper limit through to darkness, and plotting lux against voltage. To assess the impact of varying the type of light source, levels were also recorded with the same protocol using two household fluorescent tubes boxed within a diffusing cover.

Calibration factors to calculate lux for any given voltage were derived using a linear multipoint regression. This was done separately for incandescent and fluorescent light sources, based on the average sensitivity values across the three sensors. The precise recording range of each LS/Somte recording unit was calculated. The upper recording limit was the highest lux level that could be detected, within the linear range of the LS. Stability was assessed by conducting a 14 hour recording at a continuous light level of 0.1 lux to simulate an overnight PSG recording. Traces were analysed for drift (change in lux) over time.

#### Study 2: LS recordings in home and hospital settings

The LS was trialled in two “real-world” environments. Firstly, in-home PSGs[5] where the LS was positioned on the forehead of 24 able-bodied participants so that light data were not obscured by clothing or bed covers. Secondly, the LS was trialled in 12 PSGs conducted in hospital wards on patients with acute quadriplegia (the COSAQ study).[6] The LS was placed on the patient’s pillow in a position that would not be obscured by bed covers or by head movement/position change. While forehead positioning is standard for clinical use, the LS was not placed on the forehead of patients with spinal cord injury due to possible increased tactile sensitivity following injury.[7] Both studies were approved by the Austin Health Human Research Ethics Committee.

Light levels (lux) were examined to determine whether they were within the recording range of the LS, and to quantify the typical reduction at the time of Loff. The LS trace voltage value was measured using Compumedics ‘Profusion 3’ software ‘meter mode’ immediately before and after Loff. Voltages at the time of Loff were converted to lux, using the LS characteristic results from bench test. A preliminary exploration of clinical utility was also investigated, by comparison of Loff estimates with the LS (using the light sensor trace in combination with clinical interpretation of other PSG signals) and without the LS (other PSG signals only, primarily the position sensor). Estimates were made by an experienced sleep scientist blinded to the PSG order, using manual visual scoring (as per standard methodology for sleep staging and respiratory scoring [8]).

## Results

### Study 1: Light measurement characteristics of the light sensor

The maximum voltage output of the LS/Somte PSG was 250 mV. LS traces were linearly related to lux (<2% full scale non-linearity), and displayed adequate sensitivities, and trivial drift over 14 hours (Table 1). The LS recorded up to at least 350 lux for incandescent and 523 lux for fluorescent light.

**Table 1:** Light sensor (LS) trace characteristics for incandescent and fluorescent light.

	LS 1	LS 2	LS 3
***Incandescent light***			
Recording range (lux)	0–350	0–389	0–366
Sensitivity (mV/lux)	0.715	0.643	0.684
Non-linearity (full scale)	<2%	<1%	<1%
***Fluorescent light***			
Recording range (lux)	0–531	0–577	0–523
Sensitivity (mV/lux)	0.471	0.433	0.478
Non-linearity (full scale)	<1%	<1%	<2%
***Drift***			
Drift over 14 hours (lux)	0.004	0.000	0.007

### Study 2: LS recordings in home and hospital settings

**Fig 1:**
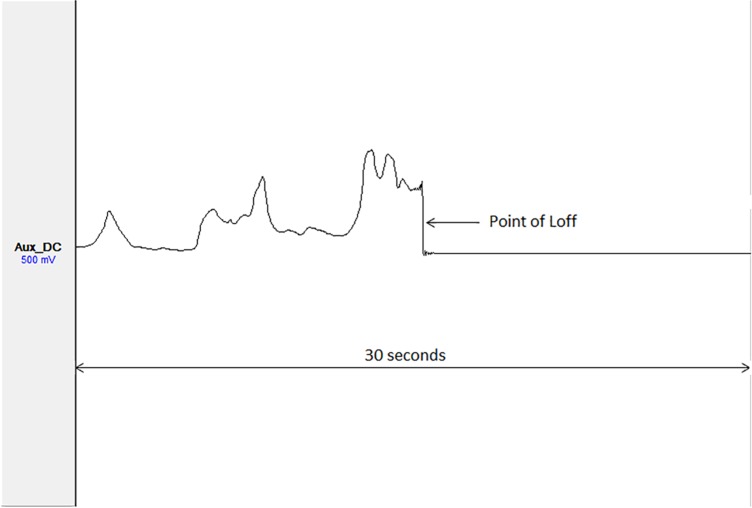
Light sensor (LS) trace example at the point of lights off (Loff).

The LS trace appearance for both home and hospital portable PSGs commonly showed heightened and variable activity prior to Loff, followed by reduced and stable activity after Loff (Table 2 and Fig 1). Light levels recorded in all home and hospital PSGs remained within the maximum recording range of the sensors.

**Table 2:** Light intensity values prior to and after lights off (Loff) in home and hospital polysomnography (PSG).

	Home	Hospital
PSGs analysed (N)	24	12
Mean (range) lux prior to Loff	70 (9–245)	78 (5–348)
Mean (range) lux after Loff	0 (0–11)	1 (0–8)
Mean (range) change in lux at time of Loff	72 (9–245)	76 (4–348)

Estimating Loff with and without the inclusion of the LS trace showed a greater than five minute change in the estimate of Loff for 25% of home PSGs (average of 13 minutes difference), and 92% of hospital PSGs (average of 18 minutes difference).

## Discussion

This study determined that the trace characteristics of a prototype LS for use in ambulatory PSG were satisfactory, that the LS recording range was suitable for the light levels observed in home and hospital ward settings, and that the LS trace showed an identifiable shift in activity at the time off Loff. These findings suggest that the LS is a valid and useful addition to ambulatory PSG. Preliminary exploration of clinical utility suggested that the LS may be particularly useful in hospital settings, but this requires further examination in controlled experimental conditions.

Unlike in a sleep laboratory, the light conditions in homes and hospital wards are variable and not easily controlled, and therefore the calibration of a LS for automated classification of light level across multiple settings is problematic. While a LS trace is objective, some important clinical considerations exist for its interpretation. Lights may be ‘off’ and ‘on’ multiple times before or after sleep is attempted. Therefore, clinical interpretation of other PSG signals is required to determine the ‘true’ clinically relevant point of Loff, where sleep is initially being attempted. The position sensor can be particularly useful, as changes from upright position to recumbent represent ‘going to bed’, as well as other signals which can inform eye movement and closure, body movement, arousal, regularity of respiration and many more. In the hospital setting, patients may be recumbent for extended periods of time, unrelated to sleep. As such, the clinical utility of the LS was highest in the hospital setting where the position sensor is less reliable as a marker of ‘going to bed’. Clinical utility data are, however, limited by lack of a gold standard light measure. Attending a sleep laboratory was not feasible for the hospital population investigated (spinal cord injury) due to the high level of medical supervision required, and not central to the aims of the current study. The different positioning of the light sensor (forehead or pillow, for able-bodied and spinal cord injury patients respectively) is a potential limitation of the current study, but is not expected to have impacted study aims or conclusions, and the LS appears to be valid for use in either position, as appropriate.

The findings of this study suggest that the LS trace provides useful additional information for the manual estimation of Loff. The position sensor, and a range of physiological PSG signals can additionally be used to determine when other activities (such as reading or watching TV) may be taking place whilst recumbent. Further, other people may control light levels in home and hospital settings, so physiological PSG signals must be used to investigate movement or wakefulness of the patient during these times. The LS trace is therefore most informative in conjunction with other PSG signals in ambulatory PSG.

Application of these findings could also extend to abbreviated home studies (such as overnight oximetry) that do not record sleep signals and could have improved diagnostic power from a more accurate Loff estimates. As total sleep time (TST) is not available for abbreviated sleep studies, total dark time, based on patient self-report, is the denominator used to calculate many indices. Self-report is an inherently poor estimate of TST and consequently can result in substantial errors in the apnoea hypopnoea index and other important PSG indices. [9, 10]

## Conclusions

Light sensors are a simple addition to ambulatory PSG providing objective and valid light data without adding significant complication or expense. Inclusion of a LS may be particularly important in hospital settings where patients are less mobile. The LS may also be useful in other portable or abbreviated overnight studies, where there is currently limited objective data. In all applications, objective light data can be used to improve the accuracy of sleep indices across research and clinical settings.

## Supporting information

S1 File Light sensor raw data output. (XLSX)
